# The rice OsNAC6 transcription factor orchestrates multiple molecular mechanisms involving root structural adaptions and nicotianamine biosynthesis for drought tolerance

**DOI:** 10.1111/pbi.12673

**Published:** 2017-01-04

**Authors:** Dong‐Keun Lee, Pil Joong Chung, Jin Seo Jeong, Geupil Jang, Seung Woon Bang, Harin Jung, Youn Shic Kim, Sun‐Hwa Ha, Yang Do Choi, Ju‐Kon Kim

**Affiliations:** ^1^ Graduate School of International Agricultural Technology and Crop Biotechnology Institute/GreenBio Science and TechnologySeoul National UniversityPyeongchangKorea; ^2^ Department of Agricultural BiotechnologySeoul National UniversitySeoulKorea; ^3^ Department of Genetic Engineering and Graduate School of BiotechnologyKyung Hee UniversityYonginKorea

**Keywords:** biotechnology, drought, NAC transcription factor, nicotianamine, rice, root

## Abstract

Drought has a serious impact on agriculture worldwide. A plant's ability to adapt to rhizosphere drought stress requires reprogramming of root growth and development. Although physiological studies have documented the root adaption for tolerance to the drought stress, underlying molecular mechanisms is still incomplete, which is essential for crop engineering. Here, we identified *OsNAC6*‐mediated root structural adaptations, including increased root number and root diameter, which enhanced drought tolerance. Multiyear drought field tests demonstrated that the grain yield of *OsNAC6* root‐specific overexpressing transgenic rice lines was less affected by drought stress than were nontransgenic controls. Genome‐wide analyses of loss‐ and gain‐of‐function mutants revealed that OsNAC6 up‐regulates the expression of direct target genes involved in membrane modification, nicotianamine (NA) biosynthesis, glutathione relocation, 3′‐phophoadenosine 5′‐phosphosulphate accumulation and glycosylation, which represent multiple drought tolerance pathways. Moreover, overexpression of *NICOTIANAMINE SYNTHASE* genes, direct targets of OsNAC6, promoted the accumulation of the metal chelator NA and, consequently, drought tolerance. Collectively, OsNAC6 orchestrates novel molecular drought tolerance mechanisms and has potential for the biotechnological development of high‐yielding crops under water‐limiting conditions.

## Introduction

Drought is a major environmental factor contributing to loss of crop yield worldwide, and in rice (*Oryza sativa*), this is due to drought‐induced phenomena such as delayed flowering time, a reduction in the number of spikelets and poor grain filling rate (Ekanayake *et al*., 1989; O'Toole and Namuco, 1983). Moreover, the proportion of agriculturally important areas with an inadequate water supply has increased substantially as a consequence of global warming and an explosive increase in human population (Mittler, 2006). Thus, the identification of plant drought tolerance mechanisms for deployment in crops is an important objective. To avoid and cope with drought stress, plants have evolved molecular mechanisms that coordinate the expression of suites of genes that protect them from drought‐induced damage, minimize loss of water and modulate their growth and development in arid environments (Shinozaki and Yamaguchi‐Shinozaki, 2007). Most drought‐inducible genes are regulated by drought‐responsive transcription factors (TFs), such as members of the AP2/ERF, MYB, bZIP and NAC families, which directly, or indirectly, regulate drought stress tolerance mechanisms (Abe *et al*., 2003; Fujita *et al*., 2004; Kang *et al*., 2002; Oh *et al*., 2009; Tran *et al*., 2004).

The NAC (NAM, ATAF and CUC) superfamily constitutes one of the largest plant‐specific TF families: 117 in *Arabidopsis thaliana*, 151 in rice, 163 in poplar (*Populus trichocarpa*) and 152 in both soybean (*Glycine max*) and tobacco (*Nicotiana tabacum*; Puranik *et al*., 2012). NAC TFs are involved in a wide range of abiotic and biotic stress responses. For example, *A. thaliana*,* AtNAC72* (*RD29*), *AtNAC109* and *AtNAC55* contribute to drought tolerance by promoting the detoxification of aldehydes in the glyoxalase pathway (Fujita *et al*., 2004; Tran *et al*., 2004), while *AtNAC2* is involved in responses to salt stress through ethylene and auxin signalling pathways (He *et al*., 2005). In rice, overexpression of *OsNAC9*,* OsNAC45*,* OsNAC52* and *OsNAC63* enhances tolerance to multiple abiotic stresses via the up‐regulation of genes involved in osmolyte production, detoxification activities, redox homeostasis and the protection of macromolecules (Hu *et al*., 2006; Redillas *et al*., 2012).

One key adaptation to drought stress involves changes in root growth and development in response to water‐deficit conditions (Sharp *et al*., 2004). Roots detect insufficient water availability in soils and release uncharacterized signals to induce resistance and/or adapt their architecture for optimal growth (Sieburth and Lee, 2010). Previous studies showed that rice inbred lines (IR20 × MGL‐2) with long and thick roots exhibit enhanced drought tolerance (Ekanayake *et al*., 1985). Moreover, overexpression of *TaNAC2* and *HRD* (*HARDY*) in *A. thaliana* promotes primary and lateral root growth and thus increasing root numbers (Karaba *et al*., 2007; Mao *et al*., 2012), while overexpression of *OsNAC5*,* OsNAC9* and *OsNAC10* in rice roots activates radial root growth (Jeong *et al*., 2010, 2013; Redillas *et al*., 2012), all of which result in enhanced drought tolerance. Recently, mechanisms involving the phytohormone auxin, regulated by *DEEPER ROOTING 1*, were shown to confer drought tolerance to rice by altering root growth angle (Uga *et al*., 2013). Thus, modification of root architecture is closely associated with drought tolerance; however, the underlying molecular mechanisms that confer root‐mediated drought tolerance are not fully understood.

*OsNAC6* is previously identified as a key regulator for rice stress responses (Nakashima *et al*., 2007; Ohnishi *et al*., 2005). Overexpression rice plants of *OsNAC6* show various stress tolerances to drought, high salinity and blast disease. The OsNAC6 acts as a transcriptional activator and up‐regulates stress‐inducible genes including lipoxygenase and peroxidase for stress tolerance (Nakashima *et al*., 2007), indicating that the *OsNAC6* is sufficient to confer stress tolerance in rice plant. Interestingly, the *OsNAC6* controls root growth at early vegetative stage through chromatin modification (Chung *et al*., 2009). It suggests a possible connection between the root structure modifications by *OsNAC6* and *OsNAC6*‐mediated drought tolerance.

In this study, we investigated the molecular mechanisms of *OsNAC6*‐mediated drought tolerance. Transgenic rice lines overexpressing *OsNAC6* under the control of either the root‐specific or the constitutive promoters showed improved drought tolerance, whereas *nac6* mutant exhibited drought susceptibility. In addition, multiyear field drought tests confirmed that root‐specific overexpression of *OsNAC6* significantly enhanced drought tolerance. We further characterized *OsNAC6*‐mediated root phenotypes related to drought tolerance. RNA‐seq and ChIP‐seq analyses led to the identification of the direct target genes of *OsNAC6*, which together constitute the *OsNAC6*‐mediated drought tolerance pathways.

## Results

### *OsNAC6* overexpression in roots is sufficient to confer drought tolerance

*OsNAC6* is a drought‐responsive TF that is also regulated by the abscisic acid as well as by low temperature and salinity stresses (Figure S1; Jeong *et al*., 2010; Nakashima *et al*., 2007). To investigate its biological roles, we designed two different constructs for *OsNAC6* overexpression in rice (Nipponbare): root‐specific *RCc3::OsNAC6* and constitutive *GOS2::OsNAC6*. To eliminate somaclonal variation, successive field selection of T_1–4_ plants was performed to identify elite lines that grew normally, without stunting. Six independent homozygous lines (#7, 24 and 38 for *RCc3::OsNAC6* and #18, 53 and 62 for *GOS2::OsNAC6*) were selected for further analysis.

**Figure 1:**
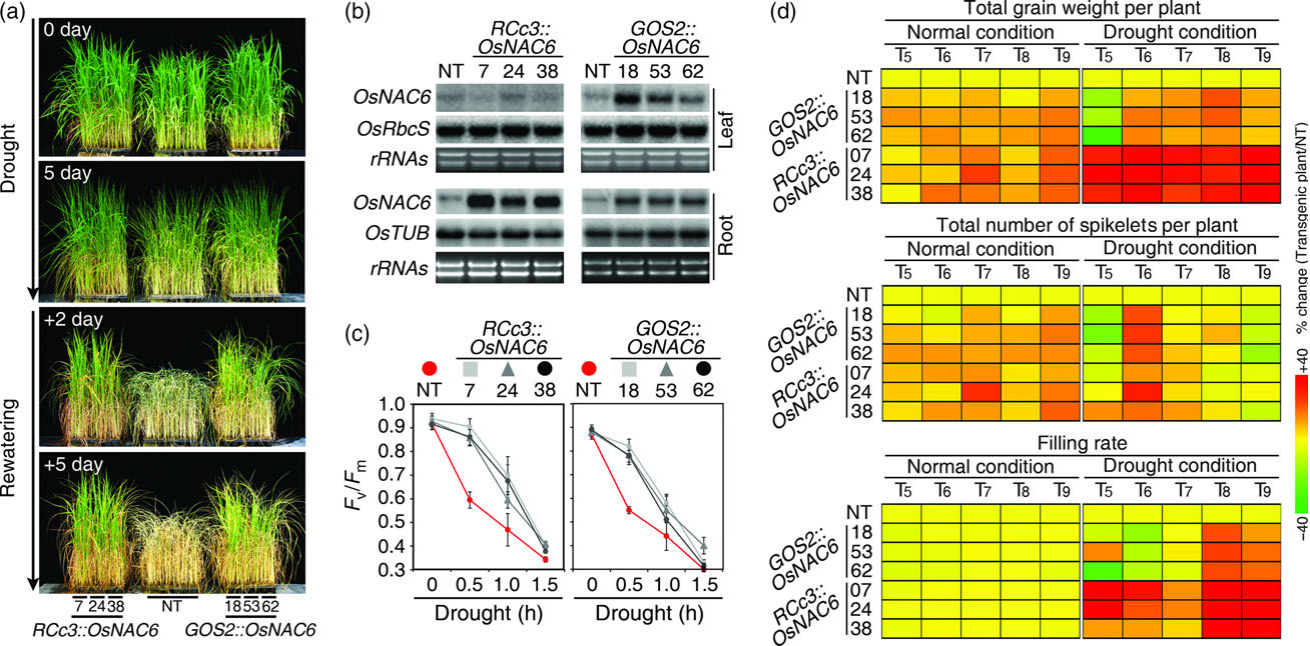
Drought tolerance of *RCc3::OsNAC6* and *GOS2::OsNAC6* transgenic plants. (a) Drought tolerance of three independent *RCc3::OsNAC6* and *GOS2::OsNAC6* lines (T_5_ generation) at a vegetative development stage. Four‐week‐old plants were exposed to drought for 5 days, followed by re‐watering. The number of days on the images indicates the duration of the drought and re‐watering. (b) RNA gel blot analysis using total RNA from leaves and roots of 4‐week‐old *RCc3::OsNAC6, GOS2::OsNAC6* and NT plants, grown under normal growth condition. *OsRbcS* and *OsTUB* were used as internal controls. (c) Photochemical efficiency test, measuring the leaf chlorophyll fluorescence (*F*_v_/*F*_m_). Values for each time point represent the mean ± SE of three‐replicate experiments (n = 30 for each genotype). (d) Heatmap of agronomic traits of three independent homozygous lines (from T_5_ to T_9_ generation) grown in the field under normal and drought conditions. Mean values for each category (n = 30 for each condition of each line) are listed in Table S1. Change (%) of total grain weight, or total number of spikelets per line in each year, was calculated as (mean value of the agronomic trait per each line/mean value of agronomic trait of NT) × 100. Filling rate (%) was (total filled grain/[total filled grain + total unfilled grain]) × 100.

To assess drought resistance, 4‐week‐old *OsNAC6* overexpressors (T_5_ generation) and nontransgenic (NT, Nipponbare) plants were subjected to progressive drought stress by withholding water for 5 days under greenhouse conditions. NT plants showed drought‐associated visual symptoms, such as leaf rolling and wilting earlier than the transgenic plants (Figure 1a). Moreover, after re‐watering, both types of *OsNAC6* overexpressors recovered better from the drought stress than the NT plants, which continued to wilt and finally died (Figure 1a). The *RCc3::OsNAC6* lines showed high levels of *OsNAC6* expression only in roots, while the *GOS2::OsNAC6* lines showed high levels of *OsNAC6* expression in both leaves and roots (Figure 1b). To independently confirm the conferred drought tolerance, we carried out a leaf chlorophyll fluorescence assay, measuring *F*_v_/*F*_m_ (*F*_v_: variable fluorescence and *F*_m_: maximum fluorescence), an indicator of photochemical efficiency of photosystem II (PSII), which can be reduced by drought stress. NT leaves exhibited a rapid decrease in *F*_v_/*F*_m_ values as early as 0.5 h after the onset of the drought treatment, while the transgenic leaves showed a delayed decrease in *F*_v_/*F*_m_ values that were ~1.5‐fold higher than those of the NT (Figure 1c), indicating that PS II of the *OsNAC6* overexpressors was less affected by drought stress. Notably, *OsNAC6* overexpression in roots alone was sufficient to confer drought tolerance during the vegetative stage of growth.

### Multiyear field tests of the *OsNAC6* overexpressors

As reproductive development is highly vulnerable to drought stress (Ekanayake *et al*., 1989; O'Toole and Namuco, 1983), and field tests represent a more informative approach to evaluate effective crop traits under agronomically relevant conditions (Nuccio *et al*., 2015), we performed drought studies of the *OsNAC6* overexpressors in a rice paddy field and focused on the reproductive development over the course of 5 years (T_5–9_ generation). *OsNAC6* overexpressors, along with NT plants, were transplanted in a paddy field in Gunwi, Korea, and grown to maturity. Yield parameters, such as total grain weight, the total number of spikelets and grain filling rate, were scored for 30 plants per transgenic event and for the NT control. Under normal growth conditions, total grain weight increased by 3%–25% in *RCc3::OsNAC6* plants and by 3%–18% in *GOS2::OsNAC6* compared with NT control plants (Figure 1d; Table S1). This increase in grain weight was mostly caused by an increase in the number of spikelets, rather than an increased filling rate (Figure 1d; Table S1), indicating that overexpression of *OsNAC6* in roots affects reproductive development, especially grain yield, under normal growth conditions.

Under drought conditions (plants exposed to intermittent drought stress at the transition stage from vegetative to reproductive development), the total grain weight of the *RCc3::OsNAC6* plants was 26%–74% greater than that of the NT controls, whereas *GOS2::OsNAC6* plants showed similar values, or only a slight increase (−32% to 22%), compared with NT plants (Figure 1d; Table S1). Given the similar levels of drought tolerance shown by *RCc3::OsNAC6* and *GOS2::OsNAC6* plants at the vegetative stage, this difference in their total grain weight under drought conditions was unexpectedly large. We determined that this was mainly due to a higher grain filling rate in the *RCc3::OsNAC6* plants than in either the NT or the *GOS2::OsNAC6* plants under drought conditions (Figure 1d; Table S1). Taken together, these results indicate that root‐specific overexpression of *OsNAC6* increases drought tolerance at the reproductive stage of growth under field drought conditions.

### *OsNAC6* expression in roots controls tiller development

**Figure 2:**
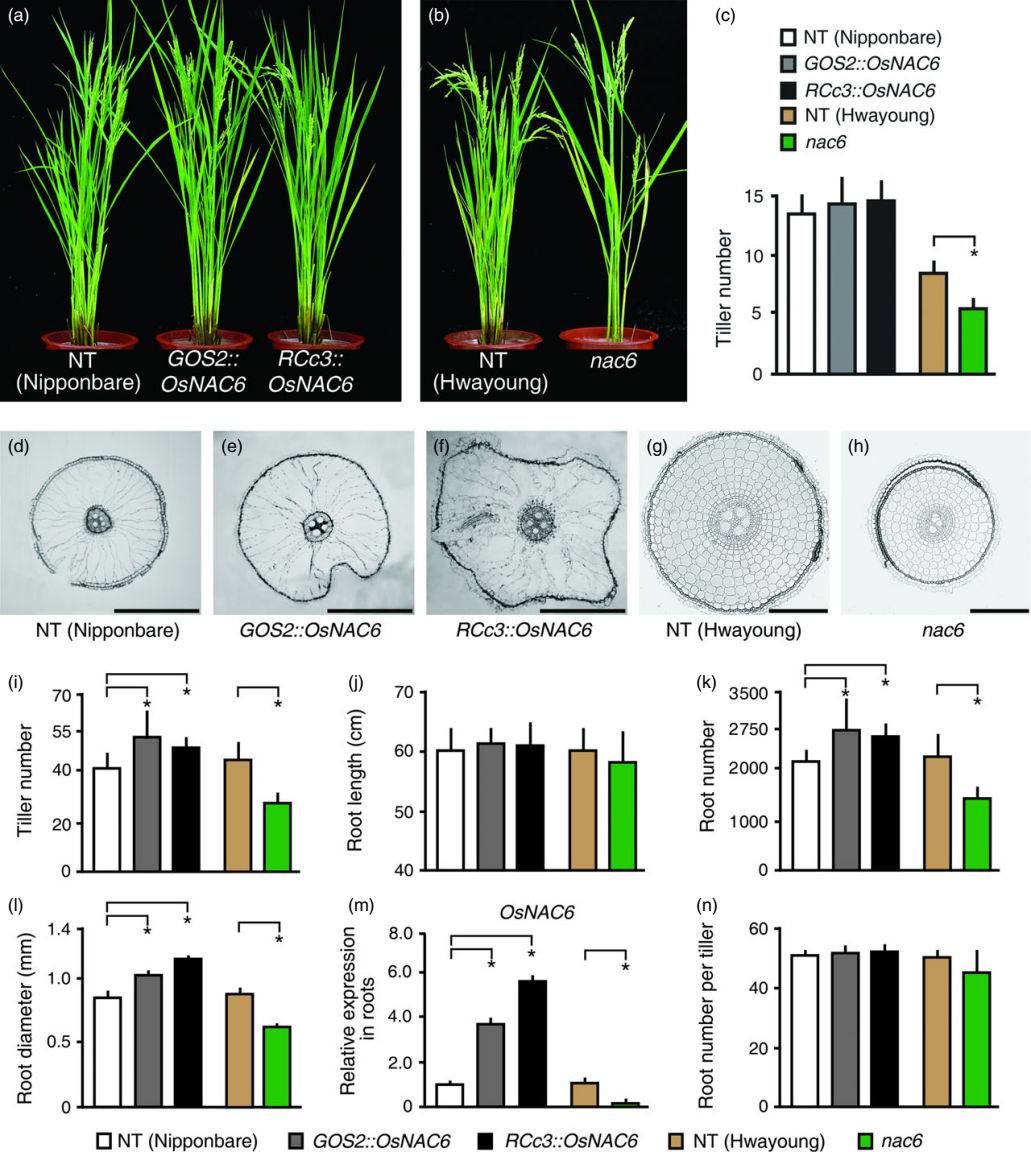
Phenotypes of *RCc3::OsNAC6*,*GOS2::OsNAC6* and *nac6* plants. (a–c) Phenotypes of 3‐month‐old *OsNAC6* overexpressors and *nac6* knockout mutants grown in a rice paddy field. Representative plants were transferred to pots for photographing. (a) *OsNAC6* overexpressors. (b) *nac6* mutants. (c) Tiller number of 3‐month‐old plants. Values shown are the mean + SD (n = 20 for each genotype). Asterisks indicate significant differences compared to NT control plants (*P *<* *0.05, Student's *t*‐test). (d–m) Phenotypes of 3‐month‐old *OsNAC6* overexpressors and *nac6* knockout mutants grown under long‐day conditions in the greenhouse. (d–f) Cross sections of root maturation zones (~30 cm from the root apex). (g, h) Cross sections of roots in the elongation zone (~3 mm from the root apex). (i–m) Quantitative analysis of phenotypes. Values shown are the mean + SD (n = 5 plants for each genotype). (i) Tiller number. (j) Root length. (k) Crown root number. (l) Root diameter (~30 cm from the root apex; 50 roots from five plants for each genotype). (m) qRT‐PCR of *OsNAC6* in 2‐week‐old roots. *UBIQUITIN 1* expression was used as an internal control. Values shown as the mean + SD of two biological replicates, each of which had two technical replicates. (n) Crown root number per tiller. Asterisks indicate significant differences compared to NT control plants (*P *<* *0.05, Student's *t*‐test). Scale bars, 500 μm in d–f and 100 μm in g and h.

To further investigate the higher spikelet number of the *OsNAC6* overexpressors under normal growth conditions, we first grew *RCc3::OsNAC6* and *GOS2::OsNAC6* plants, together with NT control, until the panicle developmental stage (approximately 3‐month‐old plants) in rice paddy fields, and counted the number of tillers. The *OsNAC6* overexpressors showed a slight increase (*P* > 0.05) in tiller number compared with NT plants (Figure 2a, c). However, as one tiller typically produces approximately 90 spikelets, the small difference in tiller number accounts for the substantially higher spikelet number in the *OsNAC6* overexpressing lines. We also evaluated *nac6*, a null mutant (Hwayoung) that has a T‐DNA insertion in *OsNAC6* (Chung *et al*., 2009), with NT (Hwayoung) plants, grown until the panicle stage in rice paddy fields. *nac6* showed reduced grain productivity, mainly due to a reduced number of spikelets and poor grain filling rate (Table S2). In addition, *nac6* produced significantly fewer (*P* < 0.05) tillers than NT plants (Figure 2b; Table S2), with averages over 2 years of 5 and 9, respectively (Figure 2c). This phenotype was rescued in complementation lines (*nac6* ^COM^), in which an *OsNAC6* genomic region was inserted into the *nac6* mutant (Figure S2a; Table S3). To further verify the role of *OsNAC6* in tiller development, we grew *OsNAC6* overexpressors and *nac6* together with NT controls (Nipponbare and Hwayoung) under long‐day growth conditions in a greenhouse, under which rice plants produce more tillers (Figure 2i). NT (Nipponbare) plants had ~40 tillers at the panicle stage, whereas *GOS2::OsNAC6* and *RCc3::OsNAC6* transgenic lines had ~54 and ~49 tillers, respectively. In addition, NT (Hwayoung) plants produced ~43 tillers, whereas *nac6* had ~25. The opposite tiller number phenotype in the *OsNAC6* overexpressors compared with *nac*6 indicates that *OsNAC6* regulates tiller development, and the root‐specific overexpression of *OsNAC6* is sufficient to promote tiller development.

### Root development is regulated by *OsNAC6*

We next compared the roots of the *OsNAC6* overexpressors and *nac6* with those of NT controls grown at the panicle stage under long‐day conditions. Root length was similar among all the genotypes (Figure 2j; Figure S2b). However, root number and diameter were significantly different between *OsNAC6* overexpressors and NT plants: NT plants had ~2,100 crown roots and an average root diameter of 0.8 mm, whereas the *OsNAC6* overexpressors had ~2,600 crown roots and a 1.1 mm root diameter (Figure 2k, l). Conversely, *nac6* had ~1,300 crown roots and an average root diameter of 0.8 mm (Figure 2k, l). We noted that large aerenchyma cells in roots of *OsNAC6* overexpressors mainly contributed to their wider root diameter (Figure 2d–f) and that cell layers in the cortex regions of *nac6* roots were substantially reduced in the elongation zones compared with NT plants (Figure 2g, h). These opposite root phenotypes were caused by expression level of the *OsNAC6* in roots of *nac6* mutants and *OsNAC6* overexpressors (Figure 2m). As root number positively correlates with tiller number (Hockett, 1986), we evaluated the average root number per tiller, which was found to be similar among all the genotypes (Figure 2n). We therefore concluded that the root number variation was caused by *OsNAC6* overexpression and the *nac6* mutation leads to abnormal tiller development.

### *OsNAC6* is necessary for rice drought tolerance

**Figure 3:**
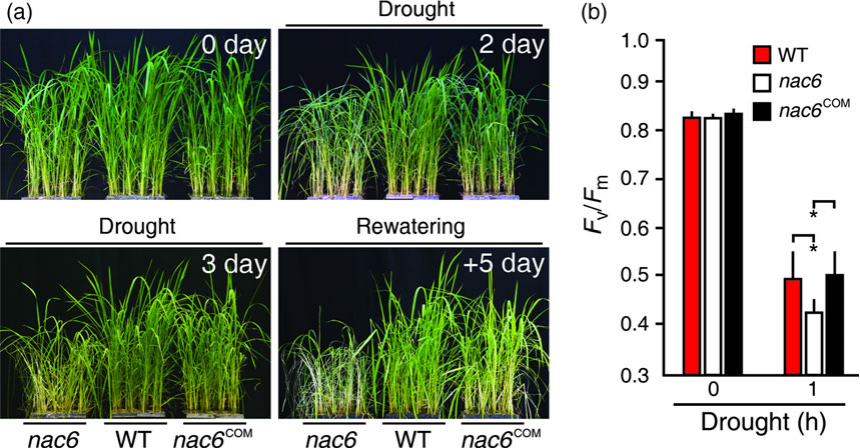
Drought susceptibility of *nac6* mutant. (a) Drought response of *nac6* and *nac6* ^COM^. Four‐week‐old plants were exposed to drought for 5 days, followed by re‐watering. The number of days on the images indicates the duration of the drought and re‐watering. (b) Photochemical efficiency (*F*_v_/*F*_m_) of 4‐week‐old *nac6* and *nac6* ^COM^ plants. Data are shown as the mean + SD of two replicates experiments (n = 20 for each genotype). Asterisks indicate significant differences compared to WT control plants (*P *<* *0.05 by Student's *t*‐test).

To test whether the *OsNAC6* is necessary for drought response, 4‐week‐old *nac6*,* nac6* ^COM^ and WT (Hwayoung) plants were subjected to progressive drought stress and we monitored drought‐induced visual symptoms (Figure 3a). Leaf rolling and wilting were detected in *nac6* earlier than *nac6* ^COM^ and WT plants (Figure 3a). Moreover, after re‐watering, both *nac6* ^COM^ and NT plants recovered better from the drought stress than the *nac6* mutants, which continued to wilt and finally died (Figure 3a). To independently confirm the drought susceptibility of *nac6* mutants, we carried out a leaf chlorophyll fluorescence assay (*F*_v_/*F*_m_). *nac6* leaves exhibited a rapid decrease in *F*_v_/*F*_m_ values at 1 h after the drought treatment, while the *nac6* ^COM^ and NT leaves showed a delayed decrease in *F*_v_/*F*_m_ values (Figure 3b), indicating that PS II of the *nac6* mutants was more damaged by drought stress and *nac6* ^COM^ plants showed normal drought response compared to WT plants. Collectively, *OsNAC6* is necessary to confer rice drought tolerance.

### *OsNAC6* is predominantly expressed in the root endodermis, pericycle and phloem

**Figure 4:**
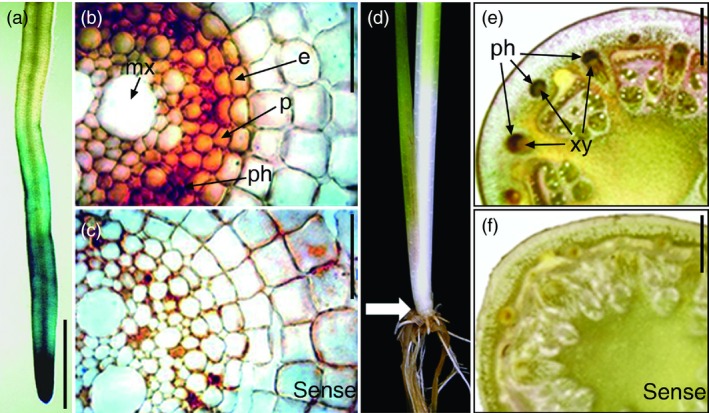
Expression patterns of *OsNAC6*. (a) Expression pattern in 4‐week‐old *OsNAC6::GUS* roots. (b, c) *In situ* hybridization analysis of *OsNAC6* in the elongation zone of 3‐month‐old roots. (b) Antisense probe. Signals were detected in the endodermis, pericycle and phloem. (c) Sense probe. (d–f) *In situ* hybridization analysis of *OsNAC6* at the base of 3‐month‐old shoots. (d) Position of a shoot cross section (arrow). (e) Antisense probe. Signals were detected in the phloem. (f) Sense probe. e, endodermis; mx, metaxylem; p, pericycle; ph, phloem; xy, xylem. Scale bars, 2 mm in a, e and f, and 100 μm in b and c.

To determine the expression patterns of *OsNAC6* in roots, we generated transgenic rice plants (*OsNAC6::GUS*) harbouring an *OsNAC6* promoter region driving expression of the β*‐GLUCURONIDASE* (*GUS*) gene. Four‐week‐old *OsNAC6::GUS* plants showed GUS activity in the root apical meristem that diminished in the root elongation zone (Figure 4a). In addition, *in situ* hybridization analysis in the elongation zone of crown roots revealed the expression of *OsNAC6* in the endodermis and pericycle, as well as the vasculature, where it was predominantly expressed in the phloem (Figure 4b, c). The phloem‐dominant *OsNAC6* expression was also observed in the base of shoots (Figure 4d–f). Expression in the endodermis and pericycle is consistent with *OsNAC6* influencing root radial growth, while expression in the vasculature suggests an association with long‐distance regulation.

### Identification of *OsNAC6*‐regulated downstream genes

**Figure 5:**
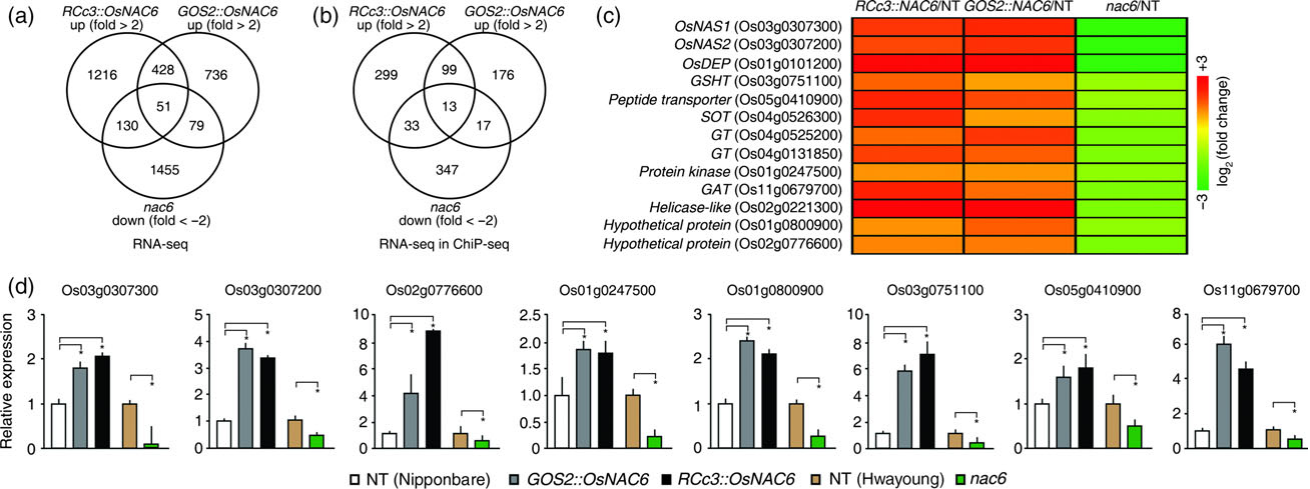
Transcriptomic analysis of *RCc3::OsNAC6*,*GOS2::OsNAC6* and *nac6* roots and a comparison between RNA‐seq and ChIP‐seq data. (a) Venn diagram of up‐regulated genes in roots of 2‐week‐old *RCc3::OsNAC6* and *GOS2::OsNAC6* relative to NT plants (cut‐off, ≥ 2.0‐fold) and down‐regulated genes in roots of *nac6* relative to NT plants (cut‐off, ≤−2.0‐fold), using RNA‐seq. (b) Venn diagram showing the number of potential genes directly up‐regulated by OsNAC6 by comparing RNA‐seq with ChIP‐seq data. (c) Thirteen high‐confidence target genes up‐regulated by OsNAC6 and a heatmap of their expression levels based on RNA‐seq data. (d) qRT‐PCR verification of genes up‐regulated by *OsNAC6*. *UBIQUITIN 1* expression was used as an internal control. Values shown as the mean + SD of two biological replicates, each of which had two technical replicates. Asterisks indicate significant differences compared to NT control plants (*P *<* *0.05, Student's *t*‐test).

To identify molecular pathways by which *OsNAC6* regulates root development and confers drought tolerance, we performed RNA‐seq analyses of five different roots from 2‐week‐old *RCc3::OsNAC6*,* GOS2::OsNAC6*,* nac6* and two NT control plants (Nipponbare and Hwayoung). As OsNAC6 functions as a transcriptional activator (Nakashima *et al*., 2007), candidate target genes regulated by OsNAC6 were identified using the following cut‐off criteria: genes with ≥2‐fold higher expression in *RCc3::OsNAC6* or *GOS2::OsNAC6* (log2 ratio ≥1.0) than in NT and genes with ≥2‐fold lower expression in *nac6* (log2 ratio ≤−1.0) than in NT. Accordingly, a total of 1,825 and 1,294 genes were up‐regulated in *RCc3::OsNAC6* and *GOS2::OsNAC6* lines, respectively, of which 479 genes were present in both sets (Figure 5a; Figure S3; Table S4). We expanded the analysis by comparing these genes with the 1,715 genes that were down‐regulated in *nac6*. Based on these RNA‐seq profiles, 51 genes were identified as potential key genes in *OsNAC6*‐mediated drought tolerance when up‐regulated in roots.

Among the 51 genes, putative direct targets were identified by considering the results of a chromatin immunoprecipitation (ChIP‐seq) analysis of roots from transgenic rice plants (*RCc3::6xmyc‐OsNAC6*; Figure S4). After removing background peaks, a total of 11,969 peaks were found to be associated with rice gene models. Among the 51 up‐regulated genes through the RNA‐seq analyses, 13 were found to be present in the ChIP‐seq profiles (Figure 5b; Table S4). The potential direct target genes included *NICOTIANAMINE SYNTHASE 1* and *NICOTIANAMINE SYNTHASE 2* (*OsNAS1* and *OsNAS2*), *DEHYDRASE‐ENLOASE PHOSPHATASE* (*OsDEP*), *GLUTATHIONE TRANSPORTER* (*GSHT*), *PEPTIDE TRANSPORTER*,* SULFOTRANSFERASE* (*SOT*), *GLUCOSYLTRANSFERASE* (*GT*), *GLYCEROL ACYLTRANSFERASE* (*GAT*) and *PROTEIN KINASE* (Figure 5c). *OsNAS1*,* OsNAS2* and *OsDEP* are key regulators of the biosynthesis of nicotianamine (NA), which acts as an Fe chelator and a potential antioxidant (Inoue *et al*., 2003; Itai *et al*., 2013; Lee *et al*., 2009, 2012). *GSHT* and *SOT* contribute to antioxidant activity through glutathione relocation and 3′‐phosphoadenosine 5′‐phosphate (PAP) biosynthesis, respectively (Klein and Papenbrock, 2004; Noctor *et al*., 2010). The accumulation of these antioxidants and PAP has been shown to alleviate drought‐induced oxidative damage (Cheng *et al*., 2015; Wilson *et al*., 2009). GAT is involved in membrane modification through lipid metabolism that affects abiotic stress tolerance (Li *et al*., 2007; Murata *et al*., 1992), while GT genes control the glycosylation of a structurally diverse range of substrates, including auxin, in association with water stress tolerance (Tognetti *et al*., 2010; Vogt and Jones, 2000). The expression of the target genes was verified by qRT‐PCR using independently isolated total RNAs from roots of the *OsNAC6* overexpressors and *nac6* (Figure 5d).

### OsNAC6 up‐regulates NA biosynthesis in roots, thereby conferring drought stress tolerance

**Figure 6:**
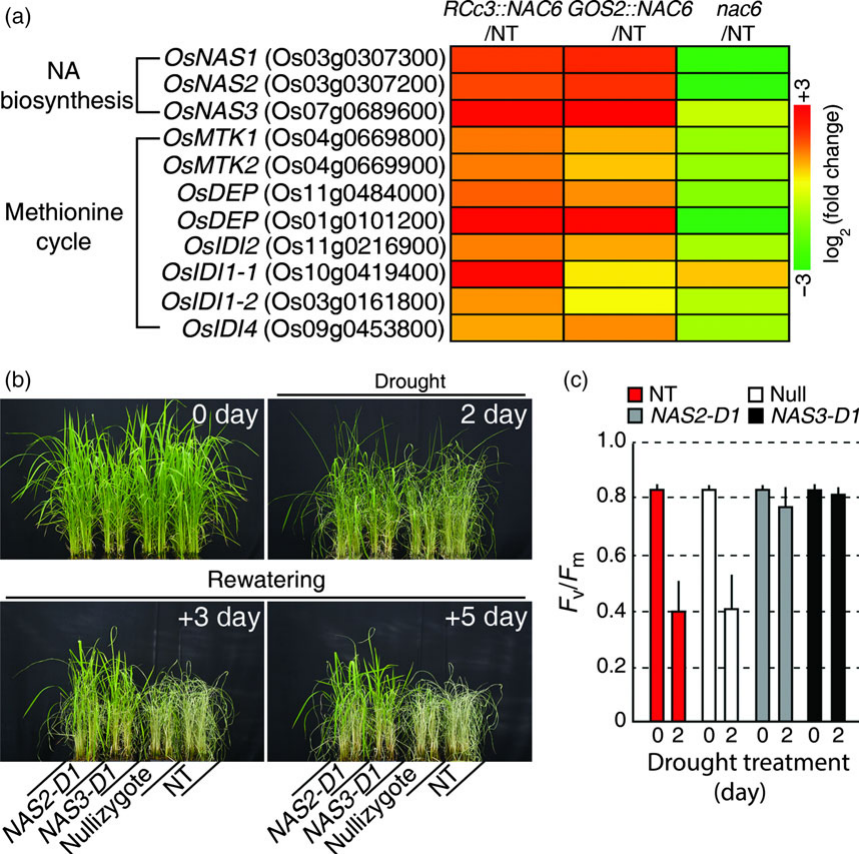
Drought tolerance of *OsNAS2‐D1* and *OsNAS3‐D1*. (a) Expression levels of genes related to NA biosynthesis and the methionine cycle regulated by *OsNAC6*, based on RNA‐seq data. (b) Drought tolerance of *OsNAS2‐D1* and *OsNAS3‐D1* plants at a vegetative developmental stage. Four‐week‐old plants were exposed to drought for 5 days, followed by re‐watering. The numbers of days on the images indicate the duration of the drought (2 day means plants that were exposed to drought stress for 2 days) and re‐watering (+3 and +5 day mean plants that were re‐watered for three and 5 days, respectively). (c) Photochemical efficiency (*F*_v_/*F*_m_) of *OsNAS2‐D1* and *OsNAS3‐D1* rice plants, grown in a greenhouse at 28–30 °C for 4 weeks and drought treated for 2 days. Each value represents the mean + SE (n = 30 for each genotype).

As *OsNAS1*,* OsNAS2* and *OsDEP* were found to be direct targets of OsNAC6, we expanded our analysis of the RNA‐seq data to include genes in the whole NA biosynthesis pathway, including the methionine (Met) cycle (Figure 6a; Table S4). This revealed that the expression of *OsNAS3*, which encodes another enzyme in NA biosynthesis, was also up‐regulated in roots of the *OsNAC6* overexpressors and down‐regulated in *nac6* roots. Similarly, the expression of *METHYLTHIORIBOSE KINASE* (*OsMTK1* and *OsMTK2*), *OsDEP*,* METHYLTHIORIBUROSE‐1‐PHOSPHATE ISOMERASE* (*OsIDI2*), *ACIREDUCTONE DIOXYGENASE* (*OsIDI1*) and *AROMATIC AMINOTRANSFERASE* (*OsIDI4*) genes, which encode key enzymes that generate *S*‐adenosyl‐Met, a NA precursor in Met cycle (Itai *et al*., 2013), were all up‐regulated in the *OsNAC6* overexpressors and down‐regulated in *nac6* (Figure 6a; Table S4). From this, we inferred that high NA accumulation in the roots of *OsNAC6* overexpressors might promote root development, leading to drought tolerance.

To determine whether increased NA accumulation in rice plants affects their responses to drought, we exposed two activation‐tagged rice lines, *OsNAS2‐D1* and *OsNAS3‐D1*, which have high levels of NA (Lee *et al*., 2009, 2012), together with nullizygotes (Null) from *OsNAS2‐D1* and NT (Dongjin) plants to drought conditions during vegetative development. NT and Null plants showed drought‐associated visual symptoms, such as leaf rolling and wilting, earlier than *OsNAS2‐D1* and *OsNAS3‐D1* plants (Figure 6b). After re‐watering, *OsNAS2‐D1* and *OsNAS3‐D1* recovered, whereas the NT and Null plants continued to wilt and finally died (Figure 6b). To further confirm the drought tolerance, we measured *F*_v_/*F*_m_ of leaves after 2 days of drought stress treatment. NT and Null leaves exhibited a sharp decrease in *F*_v_/*F*_m_ values after 2 days of drought treatment, whereas there was no significant difference in the *F*_v_/*F*_m_ values of leaves from *OsNAS2‐D1* and *OsNAS3‐D1* plants that were untreated or drought‐treated for 2 days (Figure 6c). These results suggested that *OsNAC6*‐mediated NA accumulation in roots is sufficient to confer drought tolerance.

## Discussion

We demonstrated here that the rice transcription factor *OsNAC6* is a regulator of drought tolerance pathways and represents a potentially valuable candidate for genetic engineering of drought‐tolerant high‐yielding crops. Root‐specific (*RCc3::OsNAC6*) and whole‐body (*GOS2::OsNAC6*) rice overexpression lines showed enhanced drought tolerance at the vegetative stage. Notably, only *RCc3::OsNAC6* lines showed improved drought tolerance at the reproductive stage under field drought conditions. Similar observations were made when *OsNAC5*,* OsNAC9* and *OsNAC10* were overexpressed under the control of the *RCc3* and *GOS2* promoters (Jeong *et al*., 2010, 2013; Redillas *et al*., 2012). It is possible that whole‐body overexpression of *OsNAC6* and its target genes may perturb reproductive development under drought conditions, resulting in a trade‐off in grain yield. This idea is supported by the observations that both *UBIQUITIN* promoter‐driven *OsNAC6* plants and the stress‐inducible *LIP9* promoter‐driven *OsNAC6* plants exhibit low reproductive yields (Nakashima *et al*., 2007). Furthermore, these strong promoter‐driven *OsNAC6* plants exhibit growth retardation at 14 days after germination, whereas the overexpression plants show no growth retardation at reproductive stage (Nakashima *et al*., 2007), indicating that the strong *OsNAC6* expression causes vegetative growth retardation. Collectively, these data suggest that root‐specific overexpression of *OsNAC6* represents a more effective approach than whole‐body overexpression.

Root structural adaptations to drought stresses were observed in *RCc3::OsNAC6* and *GOS2::OsNAC6* plants, which both showed higher numbers and a thicker diameter of roots, while the opposite phenotypes were observed in *nac6* mutants. Root structural modification for enhanced drought tolerance is associated with root elongation, high number of root and increased radial root growth. Overexpression of *TaNAC2* and *HRD* in *A. thaliana* or rice activates primary and lateral root elongation, which may promote the acquisition of water in deep soils (Karaba *et al*., 2007; Mao *et al*., 2012). Guidance of the roots to water sources in deep soils is regulated by *DEEPER ROOTING 1*, which modifies root growth angle (Uga *et al*., 2013). Overexpression of *HRD* also results in an increase in root number, which may enhance water uptake by increasing the total root surface area in contact with drying soils. Radial root growth, including the formation of larger aerenchyma, also enhances drought tolerance (Jeong *et al*., 2010, 2013; Redillas *et al*., 2012). In maize, the formation of root cortical aerenchyma promotes drought tolerance as it reduces the metabolic cost of soil exploration under water stress, permitting greater root growth and water acquisition from drying soil (Zhu *et al*., 2010). Moreover, the radial root growth maintains plant water potential under drought conditions (Karaba *et al*., 2007; Price *et al*., 1997). Taken together, modulation of root architecture by controlling root development can provide an effective strategy to combat to water‐deficit conditions, and our results suggest that such mechanisms occur in rice.

Our studies identified indirect and direct gene targets of OsNAC6. The RNA‐seq analyses revealed 51 up‐regulated genes by OsNAC6. After a comparison of these genes with ChIP‐seq data, we finally identified 13 up‐regulated genes as direct targets of OsNAC6, including *OsNAS1*,* OsNAS2*,* OsDEP*,* GSHT*,* SOT*,* GT* and *GAT*. These genes are involved in membrane modification, NA biosynthesis, glutathione relocation, PAP accumulation and glycosylation (Figure S5). In a previous study, OsNAC6 was found to directly regulate a peroxidase (AK104277) and a hypothetical protein (AK110725) gene, when analysed using DEX‐treated *UBIQUITIN::OsNAC6‐GR* plants (Nakashima *et al*., 2007). However, these genes were not identified through our root‐based RNA‐seq and ChIP‐seq analyses. This discrepancy may be due to differences in the tissues and promoters used in the two studies.

Drought inhibits photosynthesis due to stomatal closure, resulting in the increased production of reactive oxygen species (ROS) and the induction of drought‐mediated oxidative damage (Mittler, 2002). When ROS levels exceed the capacity of a plant to scavenge them, membrane damage can occur, due to the susceptibility of the unsaturated fatty acid components to the effects of ROS (Sharma *et al*., 2012). This was reported to be alleviated through the overexpression of *GAT* in *Nicotiana tabacum* by increasing the unsaturated fatty acid content of membranes, resulting in a stress‐tolerant phenotype (Murata *et al*., 1992). Similar phenotypes were observed when the *A. thaliana* glycerol‐3‐phosphate acyltransferase genes *GPAT4* and *GPAT8* were overexpressed, which altered the accumulation of cutin and suberin (Li *et al*., 2007). Consistent with these, *OsNAC6* overexpression was observed to mediate the *GAT* up‐regulation and enhance drought tolerance (Figure S5).

To protect themselves from drought‐mediated oxidative stress, plants produce antioxidants or osmoprotectants (Mittler, 2002; Sharma *et al*., 2012). For example, the antioxidant glutathione scavenges ROS and plays a protective role in abiotic stresses (Cheng *et al*., 2015). *OsNAC6* was found to directly up‐regulate *GSHT* expression in roots, suggesting that *OsNAC6* overexpressors activate glutathione relocation via regulation of *GSHT*, which may have alleviated drought‐mediated oxidative stress (Figure S5). In addition, sulphur metabolites play important roles in drought tolerance, and among them, PAP produced by SOT is known to accumulate during drought in *A. thaliana*, thereby contributing to drought tolerance (Chan *et al*., 2012; Wilson *et al*., 2009). Expression of *SOT* was up‐regulated in the *OsNAC6* overexpressors, suggesting that *OsNAC6* overexpressors might accumulate PAP, giving rise to drought‐tolerant phenotypes (Figure S5).

Drought can also cause an increase in Fe concentration in plants that are producing ROS, resulting in drought‐mediated oxidative damage (Moran *et al*., 1994; Price and Hendry, 1991). Fe homeostasis is influenced by the Fe chelator nicotianamine (NA), which is produced by the enzymes OsNAS1 and OsNAS2 (Inoue *et al*., 2003; Lee *et al*., 2009, 2012). In roots of plants fed with Fe, *OsNAS1* and *OsNAS2* were reported to be specifically expressed in companion and pericycle cells (Inoue *et al*., 2003). This expression pattern was similar to that of *OsNAC6*, suggesting a role for *OsNAC6* in a long‐distance Fe transport and homeostasis. Fe is an essential element for plant growth and development, especially through meristem‐specific callose deposition, which regulates cell‐to‐cell communication for root radial growth and normal root stem cell maintenance (Muller *et al*., 2015). However, excessive amount of Fe is highly toxic (Moran *et al*., 1994; Price and Hendry, 1991) and can perturb primary root elongation and lateral root formation via interaction with the auxin and ethylene signalling pathways (Giehl *et al*., 2012; Li *et al*., 2015; Ward *et al*., 2008). Despite being identified as one of the up‐regulated genes during drought conditions in *A. thaliana*, rice and wheat (Ergen *et al*., 2009; Shaik and Ramakrishna, 2013), the roles of NAS in drought tolerance mechanisms have not been reported to date. NA overproduction by OsNAC6‐mediated up‐regulation of NAS genes may promote the binding of excess Fe, thereby preventing the production of hydroxyl radicals, which consequently confers drought tolerance (Figure S5), indicating a role for NA in drought tolerance. In conclusion, overexpression of *OsNAC6* not only improves drought tolerance but also increases grain yield, further indicating its potential importance for crop improvement.

## Experimental procedures

### Plasmid construction and rice transformation

Total RNA was extracted from 2‐week‐old japonica rice roots (*Oryza sativa* cv Nipponbare), grown in a greenhouse (16‐h light/8‐h dark cycle), and used to generate total cDNAs, from which the *OsNAC6* (Os01g0884300) cDNA was amplified by PCR, using PrimeSTAR HS DNA Polymerase (Takara, Kusatsu, Japan) and the Reverse Transcription System (Promega, Madison, WI), according to the manufacturer's instructions. The primers were forward 5′‐CACCATGAGCGGCGGTCAGGACC‐3′ and reverse 5′‐CTAGAATGGCTTGCCCCAG‐3′. The PCR product was cloned into the entry vector, pENTR/SD (Invitrogen, Carlsbad, CA), and then ligated downstream of 2.2 kb of the *GOS2* (Os07g0529800) promoter in the rice transformation vector, *p700‐GOS2* (Jeong *et al*., 2010) for constitutive expression, or 1.3 kb of the *RCc3* (Os02g0662000) promoter in the rice transformation vector, *p700‐RCc3* (Jeong *et al*., 2010) for root‐specific expression, using the Gateway System (Invitrogen, Carlsbad, CA). The resulting vectors were named *GOS2::OsNAC6* and *RCc3::OsNAC6*, respectively. To generate *OsNAC6::GUS* transgenic plants, a 2‐kb promoter region (upstream region of the ATG start codon) of *OsNAC6* was amplified using PrimeSTAR HS DNA Polymerase (5′‐CTGCAGTGTGCAAACTTTCAATG TTGAC‐3′ and 5′‐GAATTCCTCTCTCCCCCTTCTCCGGT‐3′) and ligated upstream of the β*‐glucuronidase* (*GUS*) reporter gene in the rice transformation vector pCAMBIA1391Z using the *Eco*R1 and *Pst*1 restriction sites. Transgenic plants were obtained by *Agrobacterium tumefaciens* (LBA4404)‐mediated embryogenic callus (Nipponbare) transformation.

For complementation of the *nac6* mutant, 5,081 bp of a *OsNAC6* genomic fragment, including 2,240 bp of promoter (upstream region of the start ATG), 1,869 bp exons and introns (from ATG to stop codon) and 972 bp 3′ of then untranslated region, were amplified from genomic DNA of 2‐week‐old Nipponbare, using PrimeSTAR HS DNA Polymerase. The amplified genomic fragment was cloned into the rice transformation vector, pSB11 (Komori *et al*., 2007). Complementation lines were obtained by *A. tumefaciens* (LBA4404)‐mediated transformation of *nac6* (Chung *et al*., 2009) embryogenic callus.

### Stress treatments for RNA gel blot analysis

Rice (Nipponbare) seeds were germinated in soil and grown for 14 days in a greenhouse at 28–30 °C. For the drought treatment, seedlings were air‐dried under continuous light (~1000 μmol/m^2^/s), and for high‐salinity and ABA treatments, seedlings were transferred to a nutrient solution (Inoue *et al*., 2003) including 400 mm NaCl or 100 μm ABA, respectively. For low‐temperature treatments, seedlings were placed in a 4 °C cold chamber under continuous light (150 μmol/m^2^/s). Total RNA was extracted from these samples using TRIzol^®^ reagent (Invitrogen, Carlsbad, CA), and 10 μg from each sample was fractionated on a 1.2% denatured agarose gel and blotted onto a Hybond N+ nylon membrane (Amersham Bioscience, Piscataway, NJ). A radiolabeled *OsNAC6* cDNA fragment was used to probe the membrane, corresponding to a 376‐bp fragment of the 3′ untranslated region, which was generated by PCR amplification (5′‐CCTCCTCCAGGACATCCTCA‐3′ and 5′‐CGAATCAATCACCATGTACT‐3′). *OsDip1* and *OsRbcS* probes were used as markers for the stress treatments (Jeong *et al*., 2013). To determine the *OsNAC6* expression patterns and levels in the *OsNAC6* overexpressors, total RNA was extracted from the roots and leaves of three homozygous T_5_ lines of *RCc3::OsNAC6* and *GOS2::OsNAC6* plants. Ten micrograms of each total RNA sample was used for RNA gel blot analysis, as above.

### Drought stress treatment during vegetative development

Transgenic and NT control plants were germinated on Murashige and Skoog (MS) media (Duchefa, Haarlem, Netherlands) at 28 °C for 4 days, and eighteen seedlings of each transgenic lines and NT were transplanted into soil pots (4 × 4 × 6 cm; four plants per pot) and grown for 4 weeks in a greenhouse (16‐h light/8‐h dark cycle) at 28–30 °C. Each pot had the same size of holes in the bottom, and they were all placed in a single tray to synchronize watering. Drought stress was simultaneously applied to all the rice plants by first adding no water to the soil pots for 5 days and then re‐watering. Drought‐induced symptoms were monitored by imaging transgenic and NT plants at the indicated time points using a NEX‐5N camera (Sony, Tokyo, Japan).

### Measurement of chlorophyll fluorescence

*RCc3::OsNAC6*,* GOS2::OsNAC6* and NT plants were grown in greenhouse at 28–30 °C for 2 weeks. *nac6*,* nac6* ^COM^, *OsNAS2‐D1*,* OsNAS3‐D1* and NT plants were grown in greenhouse at 28–30 °C for 4 weeks. Thirty leaves from ten seedlings were collected before each stress treatment. Samples were adapted in dark conditions for 10 min. To simulate drought, the leaf discs were air‐dried for the indicated time points at 28 °C. To measure *F*_v_/*F*_m_ values, representing the activity of PSII, a PAM test was carried out with a pulse modulation fluorometer, Mini‐PAM (Walz, Effeltrich, Germany) as described previously (Redillas *et al*., 2012). The dark‐treated leaf was given a measuring light of 0.15 μmol photon m^−2^ s^−1^ for a minimal level of fluorescence and then a 0.8 s actinic light of 10 000 μmol photon m^−2^ s^−1^ for a maximal level of fluorescence.

### Phenotypic and anatomical analysis of rice roots grown under long‐day conditions

*RCc3::OsNAC6*,* GOS2::OsNAC6*,* nac6* and NT plants were transplanted to PVC tubes (1.2 m in length and 0.2 m in diameter) that were filled with natural paddy soil and placed into container filled with water. At the panicle development stage, roots were quantified and used for sectioning for anatomical analysis. The diameter of 50 individual roots from *RCc3::OsNAC6*,* GOS2::OsNAC6*,* nac6* and NT plants (five plants for each genotype) was measured. Internal root anatomy was examined using a Technovit 7100 system, as previously described (Jang *et al*., 2011) with minor modifications. Technovit saturation was carried out for 3 days. Sections (3 μm) were generated using an ultramicrotome (MTX, RMC, USA), and images were captured with an Olympus DP70 camera mounted on Olympus BX 500 light microscope.

### Agronomic trait analysis in rice paddy fields over a 5‐year period

The field experiments, including the use of fertilizers, drought treatments and analysis of agronomic traits, were as described previously (Oh *et al*., 2009). Briefly, to evaluate yield components of the transgenic plants under normal growth conditions, three independent homozygous lines from T_5_ (2009) to T_9_ (2013) for the *RCc3::OsNAC6* and *GOS2::OsNAC6* lines, together with NT, were planted in a rice paddy field at Gunwi (36˚06΄48.0˝N, 128˚38′38.0˝E), Kyungpook National University, Korea, and grown to maturity. A randomized design was employed for three replicates using three different 10 m^2^ plots. Yield parameters were scored for 30 plants per line, collected from three different plots. To evaluate the yield components of the transgenic plants under drought field conditions, we built rain‐off shelters to cover rice plants and made a semi‐field condition before drought treatment. Intermittent drought stress was applied twice during the panicle development by draining the water from the bottom of the container. When full leaf rolling was observed in the NT plants after the first drought treatment, they were irrigated overnight and subjected to a second round of drought stress until complete leaf rolling occurred again. After two drought stress treatments, the plants were irrigated until harvesting. Yield parameters were scored for 30 plants per line collected from three different plots corresponding to the drought field conditions. The results from three independent lines were compared with those of the NT controls, using one‐way ANOVA analysis.

### Quantitative real‐time PCR analysis

For quantitative real‐time PCR (qRT‐PCR) experiments, a SuperScript™ III Platinum^®^ One‐Step qRT‐PCR System (Invitrogen, Carlsbad, CA) was used to generate first‐strand cDNAs. qRT‐PCR was carried out using a Platinum^®^ SYBR^®^ Green qPCR SuperMix‐UDG (Invitrogen) and a Mx3000p Real‐Time PCR machine (Stratagene, La Jolla, CA). To validate the RNA‐seq data, total RNA was extracted from the roots of 2‐week‐old *OsNAC6* overexpressors, *nac6* and NT rice seedlings grown under normal growth conditions. Rice *UBIQUITIN 1* (Os06g0681400) was used as an internal control, and two biological replicates, each with two technical replicates, were analysed. Gene‐specific primers used for qRT‐PCR are listed in Table S5.

### *In situ* hybridization

Three‐month‐old crown roots were used for *in situ* hybridization using Technovit resin previously described (Jang *et al*., 2011), with minor modifications. Briefly, roots were incubated in FAA fixing solution (50% [v/v] ethanol, 5% [v/v] acetic acid and 3.7% [v/v] formaldehyde) for 3 h, then dehydrated and finally embedded with Technovit resin. The sections (3 μm) were made, and *OsNAC6* probes were generated by *in vitro* transcription with a DIG RNA labeling Kit (Roche, Mannheim, Germany) targeting the 376‐bp 3′ untranslated region of the *OsNAC6* mRNA, as described above.

### RNA‐seq analysis

Total RNA was extracted from the roots of 2‐week‐old *OsNAC6* overexpressors, *nac6* and NT rice seedlings grown under normal growth conditions, using an RNeasy Plant Mini Kit (Qiagen, Hilden, Germany), according to the manufacturer's instructions. A modified TruSeq method was used to construct a strand‐specific RNA‐seq library with different index primers, and libraries were sequenced using an Illumina HiSeq 2000 system in the National Instrumentation Center of Environmental Management College of Agriculture and Life Science, Seoul National University. Genes were defined as being differentially expressed if their transcript abundance was ≥2‐fold higher in *RCc3::OsNAC6* or *GOS2::OsNAC6* compared to NT (Nipponbare) or ≥2‐fold lower in *nac6* compared to NT (Hwayoung). These data can be found at http://www.ncbi.nlm.nih.gov/geo/ (Accession number: GSE81069).

### ChIP‐seq analysis

To produce *RCc3::6 × myc‐OsNAC6* plants, the coding sequence of *OsNAC6* was amplified using PrimeSTAR DNA polymerase (Takara) with the *OsNAC6*‐*B* F‐primer (GGATCCATGAGCGGCGGTCAGGACC) and the *OsNAC6*‐*N* R‐primer (GCGGCCGCG CTAGAATGGCTTGCCCCAG). After digestion of the PCR products with *Bam*HI and *Not*I, the coding sequence was ligated into the multiple cloning site of the pE3n vector (Dubin *et al*., 2008), which is flanked with a 6 *×* *myc* tag coding sequence. Finally, the *6 × myc‐OsNAC6* sequence from the *pE3n‐OsNAC6* was subcloned into the p700‐RCc3 vector (Jeong *et al*., 2010) carrying a 1.3‐kb *RCc3* promoter sequence, using the Gateway system. Chromatin immunoprecipitation (ChIP) was performed with roots of 2‐week‐old rice seedlings, as described previously (Chung *et al*., 2009). These data can be found at http://www.ncbi.nlm.nih.gov/geo/ (Accession number: GSE80986).

## Conflict of interest

No conflict of interest declared.

## Supporting information


**Figure S1** Stress‐inducible and ABA‐dependent expression of *OsNAC6*. RNA gel‐blot analyses were performed with total RNA from 2‐week old roots and leaves, showing *OsNAC6* transcript accumulation patterns in response to drought, high‐salinity, low‐temperature and ABA treatments. The blots were hybridized with an *OsDIP1* (*DEHYDRATION INDUCIBLE PROTEIN 1*) probe as a positive control for various stresses. rRNAs were used to confirm equal loading of RNAs.
**Figure S2** Phenotypes of *nac6*,* nac6* ^COM^ *, RCc3::OsNAC6*, and *GOS2::OsNAC6* plants. (a) NT, the *nac6* knockout mutant, and *nac6* ^COM^ plants grown in a rice paddy field for ~3 months. Representative plants were transferred to pots for photographing. (b) *OsNAC6* overexpressors and *nac6* knockout mutants, together with NT plants, were grown in PVC tubes under long‐day conditions in the greenhouse for ~3 months. After removing soils, images were captured using a NEX‐5N camera. Scale bar, 10 cm.
**Figure S3** Transcriptomic analysis of RNA‐seq data. Clustering of genes up‐regulated by *OsNAC6*. Each cluster corresponds to each group described in Figure 5. The indicated scale is the log_2_ value of the normalized level of gene expression.
**Figure S4** Analysis of *myc‐OsNAC6* transcripts and myc‐OsNAC6 protein in roots of *RCc3::6xmyc‐OsNAC6* transgenic plants. (a) Phenotypes of the NT control (*Oryza sativa japonica* cv. Ilmi) and *RCc3::6xmyc‐OsNAC6* lines at the reproductive stage. (b) Expression level of *myc‐OsNAC6* in *RCc3::6xmyc‐OsNAC6* lines. *UBIQUITIN 1* expression was used as an internal control. Values shown are the mean + SD of three biological replicates, each of which had two technical replicates. (c) Western blot (WB) and immunoprecipitation (IP) analyses of myc‐OsNAC6 in *RCc3::6xmyc‐OsNAC6* lines using an anti‐myc Ab.
**Figure S5** OsNAC6‐mediated drought tolerance pathways. The drought‐inducible *OsNAC6* transcription factor controls target genes that are divided into 5 categories: membrane modification, nicotianamine biosynthesis, glutathione relocation, 3′‐phophoadenosine 5′‐phosphosulfate (PAP) accumulation, and glycosylation. The OsNAC6‐mediated multiple pathways modulate root adaptation to drought stress, and drought tolerance.
**Table S1** Agronomic traits of *OsNAC6* overexpressors.
**Table S2** Agronomic traits of *nac6* under normal conditions.
**Table S3** Agronomic traits of *nac6* complementation lines (*nac6* ^COM^) under normal conditions.
**Table S5** List of gene specific primers for qRT‐PCR.

**Table S4** Genes up‐regulated by *OsNAC6* in Figure 5 and Figure S3.
