# Supplementary material for: The rice OsNAC6 transcription factor orchestrates multiple molecular mechanisms involving root structural adaptions and nicotianamine biosynthesis for drought tolerance
Source: Plant Biotechnol J. 2017 Jan 4;15(6):754–64. doi: 10.1111/pbi.12673 (PMC5425393; doi:10.1111/pbi.12673)
Supplement: Supplementary file 1 — Figure S1 Stress‐inducible and ABA‐dependent expression of OsNAC6. RNA gel‐blot analyses were performed with total RNA from 2‐week old roots and leaves, showing OsNAC6 transcript accumulation patterns in response to drought, high‐salinity, low‐temperature and ABA treatments. The blots were hybridized with an OsDIP1 (DEHYDRATION INDUCIBLE PROTEIN 1) probe as a positive control for various stresses. rRNAs were used to confirm equal loading of RNAs. Figure S2 Phenotypes of nac6, nac6 COM , RCc3::OsNAC6, and GOS2::OsNAC6 plants. (a) NT, the nac6 knockout mutant, and nac6 COM plants grown in a rice paddy field for ~3 months. Representative plants were transferred to pots for photographing. (b) OsNAC6 overexpressors and nac6 knockout mutants, together with NT plants, were grown in PVC tubes under long‐day conditions in the greenhouse for ~3 months. After removing soils, images were captured using a NEX‐5N camera. Scale bar, 10 cm. Figure S3 Transcriptomic analysis of RNA‐seq data. Clustering of genes up‐regulated by OsNAC6. Each cluster corresponds to each group described in Figure 5. The indicated scale is the log2 value of the normalized level of gene expression. Figure S4 Analysis of myc‐OsNAC6 transcripts and myc‐OsNAC6 protein in roots of RCc3::6xmyc‐OsNAC6 transgenic plants. (a) Phenotypes of the NT control (Oryza sativa japonica cv. Ilmi) and RCc3::6xmyc‐OsNAC6 lines at the reproductive stage. (b) Expression level of myc‐OsNAC6 in RCc3::6xmyc‐OsNAC6 lines. UBIQUITIN 1 expression was used as an internal control. Values shown are the mean + SD of three biological replicates, each of which had two technical replicates. (c) Western blot (WB) and immunoprecipitation (IP) analyses of myc‐OsNAC6 in RCc3::6xmyc‐OsNAC6 lines using an anti‐myc Ab. Figure S5 OsNAC6‐mediated drought tolerance pathways. The drought‐inducible OsNAC6 transcription factor controls target genes that are divided into 5 categories: membrane modification, nicotianamine biosynthesis, glutathione reloca [file PBI-15-754-s001.docx]

Plant Biotechnology Journal

Supporting Information

**The rice OsNAC6 transcription factor orchestrates multiple molecular mechanisms involving root structural adaptions and nicotianamine biosynthesis for drought tolerance**

Dong-Keun Lee, Pil Joong Chung, Jin Seo Jeong, Geupil Jang, Seung Woon Bang, Harin Jung, Youn Shic Kim, Sun-Hwa Ha, Yang Do Choi and Ju-Kon Kim

**Figure S1.** Stress-inducible and ABA-dependent expression of *OsNAC6*.

**Figure S2.** Phenotypes of *nac6*, *nac6^COM^,* *RCc3::OsNAC6*, and *GOS2::OsNAC6* plants.

**Figure S3.** Transcriptomic analysis of RNA-seq data.

**Figure S4.** Analysis of *myc-OsNAC6* transcripts and myc-OsNAC6 protein in roots of *RCc3::6xmyc-OsNAC6* transgenic plants.

**Figure S5.** OsNAC6-mediated drought tolerance pathways.

**Table S1.** Agronomic traits of *OsNAC6* overexpressors.

**Table S2.** Agronomic traits of *nac6* under normal conditions.

**Table S3.** Agronomic traits of *nac6* complementation lines (*nac6^COM^*) under normal conditions.

**Table S4.** Genes up-regulated by *OsNAC6* in Figure 4 and Figure S3.

**Table S5.** List of gene specific primers for qRT-PCR.


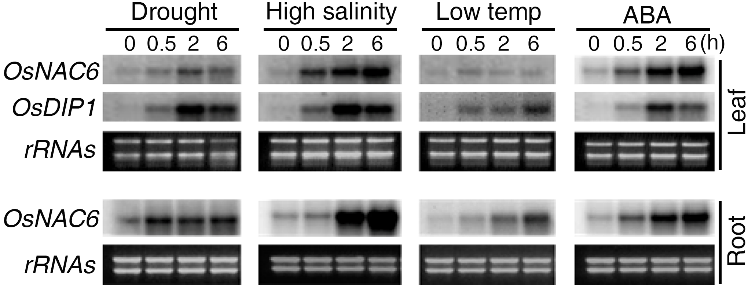


**Figure S1.** Stress-inducible and ABA-dependent expression of *OsNAC6*.

RNA gel-blot analyses were performed with total RNA from 2-week old roots and leaves, showing *OsNAC6* transcript accumulation patterns in response to drought, high-salinity, low-temperature and ABA treatments. The blots were hybridized with an *OsDIP1* (*DEHYDRATION INDUCIBLE PROTEIN 1*) probe as a positive control for various stresses. rRNAs were used to confirm equal loading of RNAs.


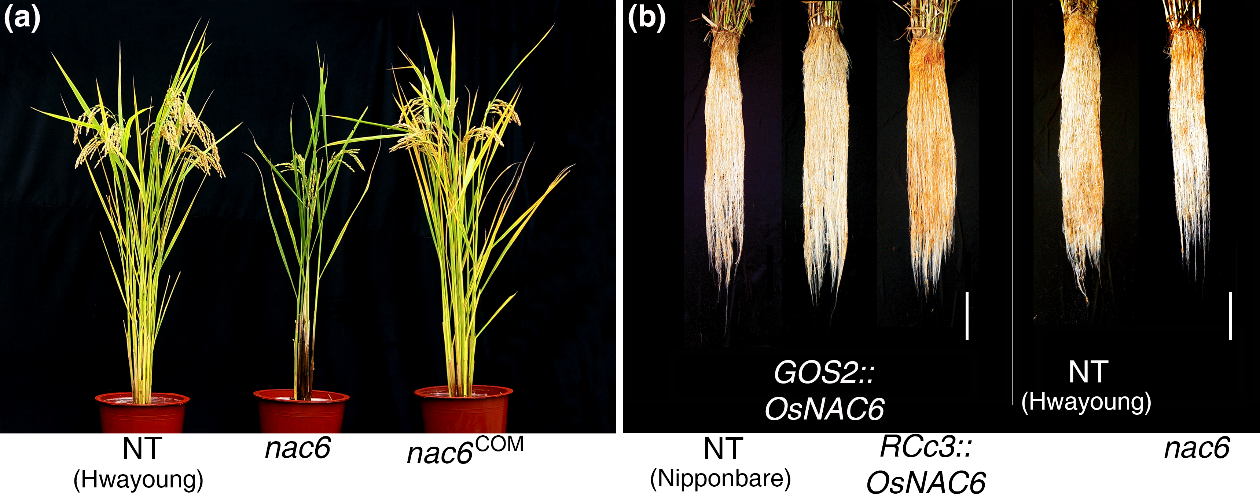


**Figure S2.** Phenotypes of *nac6*, *nac6^COM^,* *RCc3::OsNAC6*, and *GOS2::OsNAC6* plants.

(a) NT, the *nac6* knock-out mutant, and *nac6^COM^* plants were grown in a rice paddy field for ~3 months. Representative plants were transferred to pots for photographing. (b) *OsNAC6* overexpressors and *nac6* knockout mutants, together with NT plants, were grown in PVC tubes under long-day conditions in the greenhouse for ~3 months. After removing soils, images were captured using a NEX-5N camera. Scale bar, 10 cm.

**
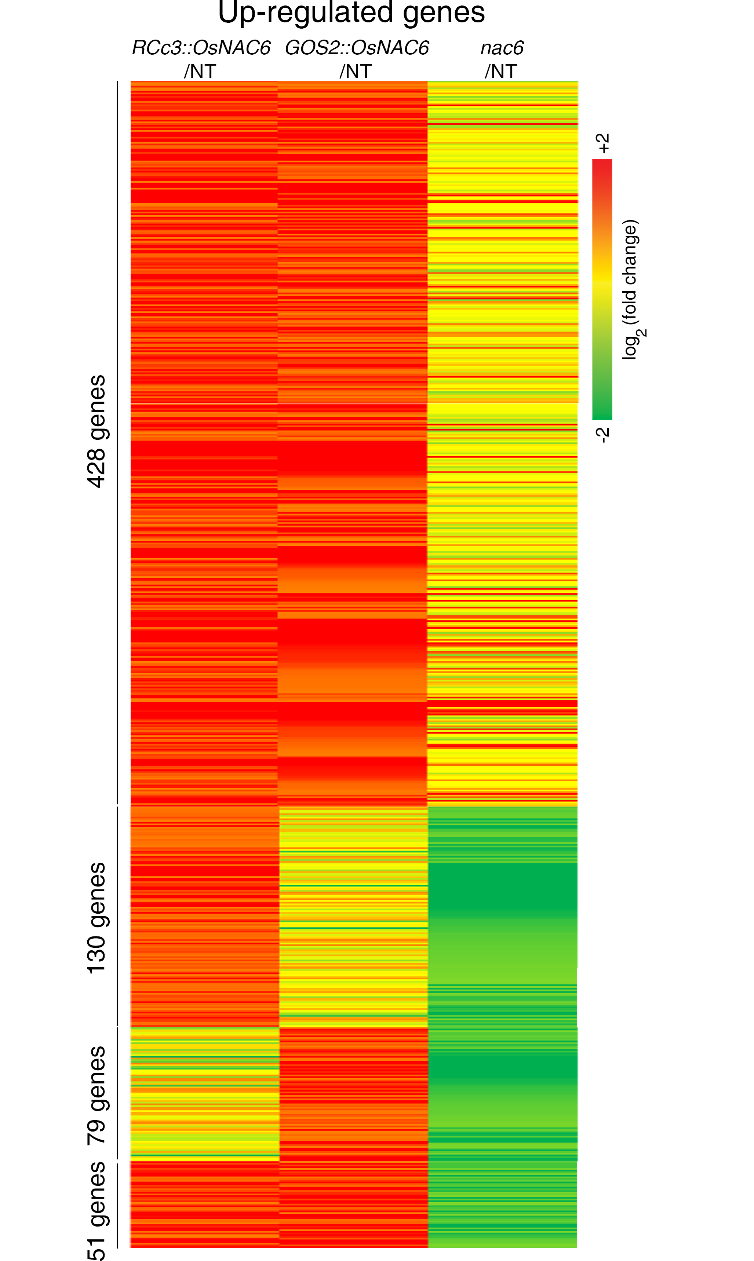
**

**Figure S3.** Transcriptomic analysis of RNA-seq data.

Clustering of genes up-regulated by *OsNAC6*. Each cluster corresponds to each group described in Figure 4. The indicated scale is the log_2_ value of the normalized level of gene expression.


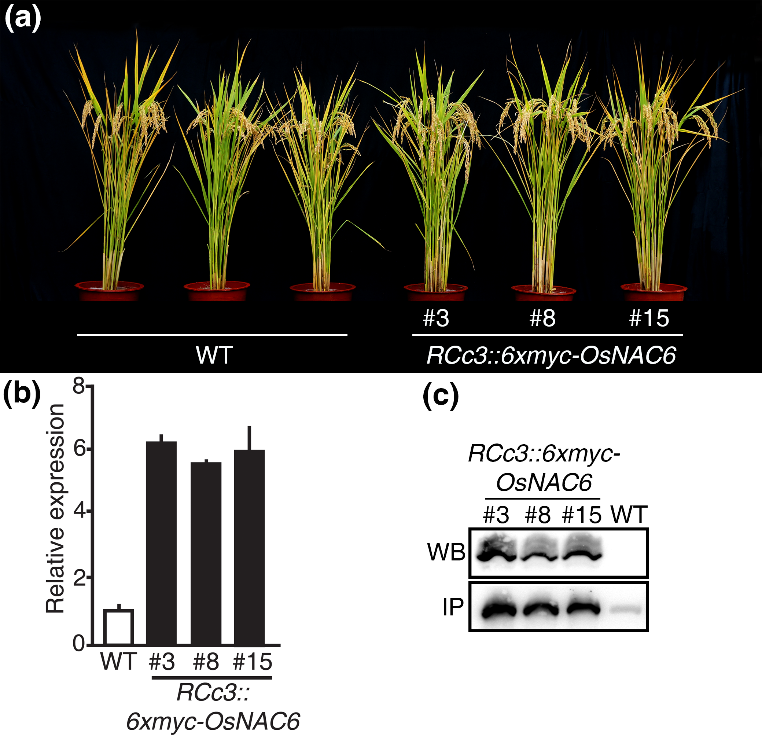


**Figure S4.** Analysis of *myc-OsNAC6* transcripts and myc-OsNAC6 protein in roots of *RCc3::6xmyc-OsNAC6* transgenic plants.

(a) Phenotypes of the NT control (*Oryza sativa japonica* cv. Ilmi) and *RCc3::6xmyc-OsNAC6* lines at the reproductive stage. (b) Expression level of *myc-OsNAC6* in *RCc3::6xmyc-OsNAC6* lines. *UBIQUITIN 1* expression was used as an internal control. Values shown are the mean + SD of three biological replicates, each of which had two technical replicates. (c) Western blot (WB) and immunoprecipitation (IP) analyses of myc-OsNAC6 in *RCc3::6xmyc-OsNAC6* lines using an anti-myc Ab.


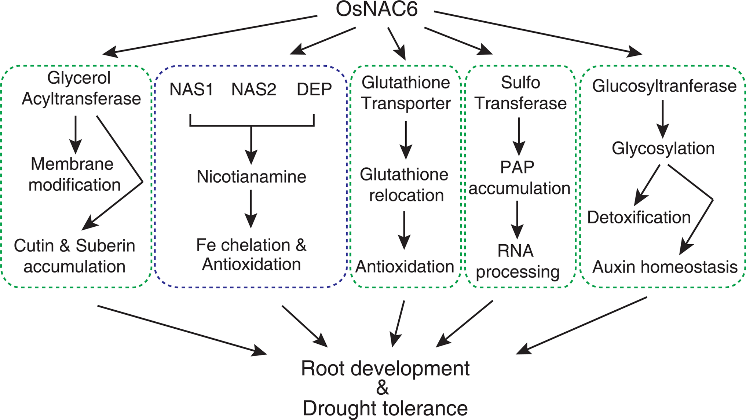


**Figure S5.** OsNAC6-mediated drought tolerance pathways.

The drought-inducible *OsNAC6* transcription factor controls target genes that are divided into 5 categories: membrane modification, nicotianamine biosynthesis, glutathione relocation, 3’-phophoadenosine 5’-phosphosulfate (PAP) accumulation, and glycosylation. The OsNAC6-mediated multiple pathways modulate root adaptation to drought stress, and drought tolerance.

**Table S1.** Agronomic traits of *OsNAC6* overexpressors.

| **Table S1.** Agronomic traits of *OsNAC6* overexpressors. | | | | | | | | | | | | | | | | | | | | |  |
| --- | --- | --- | --- | --- | --- | --- | --- | --- | --- | --- | --- | --- | --- | --- | --- | --- | --- | --- | --- | --- | --- |
| Genotypes | | 2009 (T_5_) | | | 2010 (T_6_) | | | | 2011 (T_7_) | | | | 2012 (T_8_) | | | | 2013 (T_9_) | | | |  |
|  |  | N^a^ | D^b^ | | N^a^ | | D^b^ | | N^a^ | | D^b^ | | N^a^ | | D^b^ | | N^a^ | | D^b^ | |  |
| Total grain weight (g) | | | | | | | | | | | | | | | | | | | | |  |
| Nipponbare | | 20.80 | 8.72 | | 27.82 | | 10.09 | | 20.17 | | 9.67 | | 27.24 | | 12.25 | | 21.51 | | 11.25 | |  |
| *GOS2::NAC6-18* | | **22.71** | 7.52 | | 29.73 | | 11.22 | | **22.81** | | 11.09 | | 28.22 | | **15.03** | | 24.11 | | 12.62 | |  |
| *P Value*^c^ | | 0.026 | 0.139 | | 0.252 | | 0.398 | | 0.006 | | 0.264 | | 0.471 | | 0.037 | | 0.072 | | 0.405 | |  |
| *GOS2::NAC6-53* | | **23.89** | 7.75 | | 31.36 | | 11.94 | | **23.39** | | 11.32 | | **30.88** | | 14.90 | | **25.43** | | 12.50 | |  |
| *P Value*^c^ | | 0.000 | 0.211 | | 0.052 | | 0.236 | | 0.001 | | 0.131 | | 0.042 | | 0.081 | | 0.035 | | 0.351 | |  |
| *GOS2::NAC6-62* | | **22.81** | **5.96** | | **32.14** | | 11.75 | | **22.74** | | 10.81 | | **30.99** | | 14.09 | | **25.37** | | 12.21 | |  |
| *P Value*^c^ | | 0.003 | 0.001 | | 0.025 | | 0.326 | | 0.009 | | 0.231 | | 0.007 | | 0.160 | | 0.017 | | 0.431 | |  |
| *RCc3::NAC6-7* | | 21.70 | **11.44** | | 31.21 | | **15.06** | | **24.43** | | **13.13** | | 29.17 | | **16.03** | | **26.01** | | **15.77** | |  |
| *P Value*^c^ | | 0.227 | 0.003 | | 0.076 | | 0.001 | | 0.000 | | 0.001 | | 0.168 | | 0.029 | | 0.003 | | 0.006 | |  |
| *RCc3::NAC6-24* | | **22.70** | **12.83** | | 30.12 | | **17.62** | | **26.28** | | **13.11** | | **30.02** | | **15.92** | | **26.40** | | **16.95** | |  |
| *P Value*^c^ | | 0.011 | 0.000 | | 0.190 | | 0.000 | | 0.000 | | 0.003 | | 0.049 | | 0.026 | | 0.002 | | 0.000 | |  |
| *RCc3::NAC6-38* | | 21.51 | **11.29** | | **33.17** | | **14.01** | | **24.46** | | 12.20 | | 29.42 | | **16.27** | | **25.92** | | **16.13** | |  |
| *P Value*^c^ | | 0.328 | 0.022 | | 0.004 | | 0.005 | | 0.000 | | 0.084 | | 0.131 | | 0.007 | | 0.001 | | 0.009 | |  |
| Total number of spikelet | | | | | | | | | | | | | | | | | | | | |  |
| Nipponbare | 891.27 | | | 869.63 | | 1468.23 | | 1089.50 | | 1092.77 | | 1344.17 | | 1282.07 | | 1244.00 | | 936.63 | | 1213.83 | |
| *GOS2::NAC6-18* | 923.50 | | | 757.04 | | 1415.23 | | **1340.18** | | **1220.93** | | 1374.46 | | 1313.07 | | 1306.00 | | **1062.03** | | 1138.44 | |
| *P Value*^c^ | 0.333 | | | 0.058 | | 0.512 | | 0.005 | | 0.007 | | 0.653 | | 0.634 | | 0.189 | | 0.039 | | 0.357 | |
| *GOS2::NAC6-53* | **980.37** | | | **649.20** | | 1541.23 | | **1374.28** | | **1221.07** | | **1407.08** | | **1429.30** | | **1381.83** | | **1104.66** | | 1135.90 | |
| *P Value*^c^ | 0.004 | | | 0.001 | | 0.397 | | 0.006 | | 0.012 | | 0.459 | | 0.043 | | 0.006 | | 0.027 | | 0.322 | |
| *GOS2::NAC6-62* | **1018.20** | | | 817.00 | | **1680.87** | | **1334.65** | | **1232.47** | | 1467.21 | | **1468.63** | | 1300.17 | | **1081.80** | | **1047.14** | |
| *P Value*^c^ | 0.001 | | | 0.381 | | 0.025 | | 0.009 | | 0.005 | | 0.155 | | 0.003 | | 0.231 | | 0.033 | | 0.031 | |
| *RCc3::NAC6-7* | **981.23** | | | 832.58 | | 1673.20 | | 1248.50 | | **1256.50** | | 1483.88 | | 1353.27 | | 1204.59 | | 1049.50 | | 1193.50 | |
| *P Value*^c^ | 0.003 | | | 0.542 | | 0.054 | | 0.074 | | 0.006 | | 0.101 | | 0.267 | | 0.414 | | 0.053 | | 0.871 | |
| *RCc3::NAC6-24* | **969.60** | | | 895.29 | | 1544.17 | | **1429.61** | | **1392.93** | | 1429.96 | | 1399.17 | | 1226.00 | | **1103.27** | | 1229.52 | |
| *P Value*^c^ | 0.016 | | | 0.691 | | 0.370 | | 0.002 | | 0.000 | | 0.248 | | 0.077 | | 0.713 | | 0.011 | | 0.847 | |
| *RCc3::NAC6-38* | 945.10 | | | **1003.72** | | **1677.90** | | **1297.61** | | **1242.07** | | **1532.33** | | 1373.67 | | 1313.17 | | **1127.79** | | 1134.79 | |
| *P Value*^c^ | 0.088 | | | 0.042 | | 0.021 | | 0.018 | | 0.002 | | 0.036 | | 0.164 | | 0.143 | | 0.013 | | 0.341 | |
| Grain filling rate (%) | | | | | | | | | | | | | | | | | | | | |  |
| Nipponbare | | 91.98 | 49.19 | | 82.74 | | 47.62 | | 86.51 | | 50.03 | | 89.23 | | 40.77 | | 86.88 | | 44.29 | |  |
| *GOS2::NAC6-18* | | **89.48** | 46.37 | | 83.56 | | 40.57 | | 8661 | | 50.91 | | 90.30 | | **50.08** | | 88.65 | | 50.39 | |  |
| *P Value*^c^ | | 0.001 | 0.489 | | 0.407 | | 0.128 | | 0.928 | | 0.817 | | 0.058 | | 0.026 | | 0.064 | | 0.063 | |  |
| *GOS2::NAC6-53* | | **90.39** | **57.17** | | 81.49 | | 43.09 | | **89.02** | | **52.34** | | 90.40 | | **51.04** | | 88.17 | | **52.81** | |  |
| *P Value*^c^ | | 0.028 | 0.049 | | 0.242 | | 0.350 | | 0.012 | | 0.429 | | 0.103 | | 0.017 | | 0.173 | | 0.023 | |  |
| *GOS2::NAC6-62* | | **89.13** | **35.60** | | 81.23 | | 43.27 | | 87.41 | | 48.67 | | **91.42** | | **50.36** | | 85.50 | | **53.11** | |  |
| *P Value*^c^ | | 0.000 | 0.003 | | 0.275 | | 0.380 | | 0.444 | | 0.617 | | 0.000 | | 0.023 | | 0.152 | | 0.032 | |  |
| *RCc3::NAC6-7* | | 91.24 | **69.65** | | 85.00 | | **63.84** | | **88.86** | | **57.51** | | **90.77** | | **60.21** | | 88.64 | | **67.20** | |  |
| *P Value*^c^ | | 0.408 | 0.000 | | 0.057 | | 0.002 | | 0.030 | | 0.003 | | 0.025 | | 0.000 | | 0.088 | | 0.000 | |  |
| *RCc3::NAC6-24* | | 92.88 | **70.41** | | 84.30 | | 58.22 | | 88.14 | | **58.37** | | **90.93** | | **57.81** | | **89.28** | | **69.25** | |  |
| *P Value*^c^ | | 0.173 | 0.000 | | 0.142 | | 0.059 | | 0.150 | | 0.002 | | 0.025 | | 0.000 | | 0.005 | | 0.000 | |  |
| *RCc3::NAC6-38* | | 91.16 | 55.84 | | **85.20** | | 54.08 | | 88.42 | | 53.79 | | **90.98** | | **58.43** | | 87.02 | | **67.17** | |  |
| *P Value*^c^ | | 0.284 | 0.155 | | 0.009 | | 0.166 | | 0.098 | | 0.227 | | 0.008 | | 0.000 | | 0.864 | | 0.000 | |  |
| ^a^Normal growth conditions; ^b^Drought growth conditions; ^c^One-way ANOVA. Each parameter value represents the mean (n = 30).  Numbers in boldface indicate a significant difference (*P* < 0.05). | | | | | | | | | | | | | | | | | | | | |  |

**Table S2.** Agronomic traits of *nac6* under normal conditions.

| **Table S2.** Agronomic traits of *nac6* under normal conditions. | | | | | | | |
| --- | --- | --- | --- | --- | --- | --- | --- |
| Year | Genotype | Culm length (cm) | Panicle length (cm) | Number of tillers (per rice) | Total spikelets (per rice) | Filling rate (%) | Total grain weight (g) |
| 2012 | NT^a^ | 69.98 | 21.36 | 7.40 | 826.60 | 90.25 | 18.41 |
|  | *nac6* | 71.88 | 21.36 | **5.11** | 668.78 | **75.09** | **11.44** |
|  | *p-value*^b^ | 0.76 | 0.90 | 0.01 | 0.21 | 0.00 | 0.01 |
| 2013 | NT^a^ | 78.78 | 23.25 | 10.00 | 1145.93 | 92.02 | 29.00 |
|  | *nac6* | **70.35** | 22.79 | **5.43** | **640.61** | **71.83** | **16.92** |
|  | *p-value*^b^ | 0.00 | 0.18 | 0.00 | 0.00 | 0.00 | 0.00 |
| ^a^NT, Hwayoung. ^b^One-way ANOVA. Each parameter value represents the mean (n = 30).  Numbers in boldface indicate a significant difference (*P* < 0.05) | | | | | | | |

**Table S3.** Agronomic traits of *nac6* complementation lines (*nac6^COM^*) under normal conditions.

| **Table S3.** Agronomic traits of *nac6* complementation lines (*nac6^COM^*) under normal conditions. | | | | | | |
| --- | --- | --- | --- | --- | --- | --- |
| Genotype | Culm length (cm) | Panicle length (cm) | Number of tillers (per rice) | Total spikelets (per rice) | Filling rate (%) | Total grain weight (g) |
| NT^a^ | 76.71 | 22.60 | 14.24 | 1309 | 84.16 | 28.83 |
| *nac6^COM^*-1 | 74.12 | 21.23 | 13.21 | 1219 | 86.63 | 27.61 |
| *p-value*^b^ | 0.54 | 0.45 | 0.65 | 0.56 | 0.32 | 0.25 |
| *nac6^COM^*-2 | 79.98 | 23.18 | 13.51 | 1333 | 83.83 | 27.51 |
| *p-value*^b^ | 0.35 | 0.26 | 0.84 | 0.74 | 0.14 | 0.23 |
| ^a^NT, Hwayoung. ^b^One-way ANOVA. Each parameter value represents the mean (n = 30).  Numbers in boldface indicate a significant difference (*P* < 0.05) | | | | | | |

**Table S4.** Genes up-regulated by *OsNAC6* in Figure 4 and Figure S3.

**An Excel File (up-loaded separately).**

**Table S5** List of gene specific primers for qRT-PCR.

|  | | |
| --- | --- | --- |
| **Table S5.** List of gene specific primers for qRT-PCR. | | |
| Gene ID | Forward primers | Reverse primers |
| Os01g0884300 | TGCTCGGAGCAGGTGCTGTC | GGCTTGCCCCAGTACATGAGG |
| Os01g0101200 | GTAGCAGGGCATCAGAGTTGAC | TGGTATCACACCCCTTGCACTG |
| Os01g0247500 | CTGGAGCAAGCCAAGAACACTAC | TCTTTGGTAGCTCGATCGCCAG |
| Os01g0800900 | TCGAGATGATGGACGCGAAG | ATCGTCGATGCTCACGTACC |
| Os02g0776600 | ACGCCATCTACCACTCCCTCG | GCGACGTCTCCTGTTGACTCC |
| Os03g0307200 | ATCGGCCGGCTGAAACATTATTGG | AAACACGGAATGCATTGGCACGG |
| Os03g0307300 | CTAACAGCCGGACGATCGAAAGG | TTACACTGGCTTACAGGGTCCTC |
| Os03g0751100 | TGTTCAAGTACCGCAAGGGGTGG | CCTACATACACCACCGTGTGCAG |
| Os11g0679700 | GACCCGCTCTACTACATCGCC | GGCGAGCATGAGGTACTTGTCC |
| Os06g0681400 | ATGGAGCTGCTGCTGTTCTA | TTCTTCCATGCTGCTCTACC |
